# Thiophosphate Analogs of Coenzyme A and Its Precursors—Synthesis, Stability, and Biomimetic Potential

**DOI:** 10.3390/biom12081065

**Published:** 2022-08-01

**Authors:** Christian Löcherer, Elif Tosun, Hannah Backes, Andres Jäschke

**Affiliations:** Institute of Pharmacy and Molecular Biotechnology, Heidelberg University, Im Neuenheimer Feld 364, 69120 Heidelberg, Germany; c.loecherer@gmx.de (C.L.); e.tosun@gmx.net (E.T.); hannah.backes@t-online.de (H.B.)

**Keywords:** CoA, PPanSH, phosphorothioate, CoA biosynthesis, CoA degradation, Nudt7, CoA deficiency, HoPan, PKAN, NBIA

## Abstract

Coenzyme A (CoA) is ubiquitous and essential for key cellular processes in any living organism. Primary degradation of CoA occurs by enzyme-mediated pyrophosphate hydrolysis intracellularly and extracellularly to form adenosine 3’,5’-diphosphate and 4’-phosphopantetheine (PPanSH). The latter can be recycled for intracellular synthesis of CoA. Impairments in the CoA biosynthetic pathway are linked to a severe form of neurodegeneration with brain iron accumulation for which no disease-modifying therapy is available. Currently, exogenous administration of PPanSH is examined as a therapeutic intervention. Here, we describe biosynthetic access to thiophosphate analogs of PPanSH, 3′-dephospho-CoA, and CoA. The stabilizing effect of thiophosphate modifications toward degradation by extracellular and peroxisomal enzymes was studied in vitro. Experiments in a CoA-deficient cell model suggest a biomimetic potential of the PPanSH thiophosphate analog P_S_PanSH (C1). According to our findings, the administration of P_S_PanSH may provide an alternative approach to support intracellular CoA-dependent pathways.

## 1. Introduction

Coenzyme A (CoA) plays a pivotal role in various cellular processes related to metabolism, biotransformation, energy production, signal transduction, epigenetics, and redox homeostasis [1,2,3,4]. Since CoA itself is neither cell membrane permeable nor serum stable, the molecule is mainly produced from extracellular pantothenate, also known as vitamin B_5_ [5]. In mammals, pantothenate is actively absorbed into the cell via the sodium-dependent multivitamin transporter and converted to CoA by a cascade involving five reactions and four enzymes [6]. Mutations in the mitochondrial isoform of the first enzyme, pantothenate kinase 2 (PANK2), result in substantial CoA deficiency associated with brain iron accumulation and severe symptoms of neurodegeneration [7]. Three main strategies are currently being investigated for the treatment of this genetic disorder, termed pantothenate kinase-associated neurodegeneration (PKAN): iron chelation, activation of alternative PANK isoforms, and supplementation with CoA or CoA precursor molecules (see Thakur et al., 2021 for a review on current PKAN therapies) [8,9]. One promising candidate is the PANK2-independent CoA precursor 4′-phosphopantetheine (PPanSH) that is currently being investigated in a phase 2 clinical trial (NCT04182763) [10]. Endogenous PPanSH is formed during pyrophosphate hydrolysis of CoA, which occurs extracellularly but also within mitochondria and peroxisomes [5,11,12,13]. PPanSH is converted back to CoA by the enzyme CoA synthase (COASY) at the outer mitochondrial membrane [14]. Passive membrane diffusion is assumed to be the major cellular uptake mechanism of exogenous PPanSH, despite its polarity due to the negatively charged phosphate group [5].

Oxygen-to-sulfur exchange in phosphate groups of biomolecules such as NAD^+^, m^7^G cap structures, or in the backbone of ribonucleic acids has been shown to result in increased lipophilicity as well as increased stability toward nucleases and pyrophosphatases, with a strong dependence on the stereochemical configuration of the chiral phosphorus of the thiophosphate moiety [15,16,17].

In this manuscript, we test an enzymatic approach for the synthesis of thiophosphate analogs of PPanSH as well as of its cellular successors, 3′-dephospho-CoA (dpCoA) and CoA. Selected candidates are evaluated for their stability toward (pyro)phosphatases in vitro and the behavior in cellulo in comparison to PPanSH.

## 2. Materials and Methods

All reagents were purchased from Sigma Aldrich (Schnelldorf, Germany) unless other-wise stated. Experimental details are provided in the Supplementary Information.

### 2.1. Protein Expression and Purification

All proteins were expressed in *Escherichia coli* (*E. coli)* BL21(DE3) that contained plasmids for the respective proteins and an additional 6x His-tag. pET28a-Ec.coaA was a gift from Erick Strauss (Addgene plasmid # 50386). pET28a-Ec.coaD (pESC106) was a gift from Tadhg Begley and Erick Strauss (Addgene plasmid # 50388). pET28a-Ec.coaE (pESC124) was a gift from Tadhg Begley and Erick Strauss (Addgene plasmid # 50390). NUDT7 (NUDT7A-c002) was a gift from Nicola Burgess-Brown (Addgene plasmid # 98235). The proteins pantothenate kinase (PanK), phosphopantetheine adenylyltransferase (PPAT), and dpCoA kinase (DPCK) were purified by immobilized metal affinity chromatography. Human Nudix-type hydrolase Nudt7 was additionally purified by size-exclusion chromatography. Experimental details are described in the Supplementary Information.

### 2.2. Enzymatic Synthesis of Thiophosphate Analogs of PPanSH, dpCoA, and CoA

In general, substrates were incubated with tris(2-carboxyethyl)phosphine (TCEP), and *E. coli* PanK, *E. coli* PPAT, or *E. coli* DPCK in 20 mM KCl, 20 mM MgCl_2_, 300 mM Tris HCl (pH 7.5 or pH 8) at 37 °C and 300 rpm shaking for 15 h. Purification was performed by preparative reversed-phase high-performance liquid chromatography (RP-HPLC). Products were obtained as triethylammonium (TEA) salts and analyzed by high-resolution mass spectrometry (HR-MS) and ^31^P nuclear magnetic resonance (NMR) spectroscopy. Compound-specific experimental details are described in the Supplementary Information.

### 2.3. Synthesis of Calcium Hopantenate (HoPan)

The synthesis was performed as previously published [18]. Experimental details are described in the Supplementary Information.

### 2.4. Serum Stability of C1

C1 was incubated in 80% dialyzed fetal bovine serum (FBS, ThermoFisher Scientific, Dreieich, Germany) at 37 °C. ^31^P NMR spectra were recorded every 2 h for 56 h in total. Experimental details are described in the Supplementary Information. 

### 2.5. Pyrophosphate Hydrolysis in Fetal Bovine Serum (FBS)

dpCoA, C2a, C3a, C3b, CoA, or C4 were incubated in 75% pre-warmed FBS (ThermoFisher Scientific) at 37 °C and 300 rpm shaking for defined time intervals. Then, TCEP was added to reduce disulfides. After protein denaturation at 95 °C for 5 min and 10 kDa cutoff-filtration, the thiol compounds were incubated with the fluorescent dye 7-diethylamino-3-(4-maleimidophenyl)-4-methyl-coumarin (CPM, Abcam, Cambridge, UK, stock solution in dimethyl sulfoxide). Thiol-CPM conjugates were analyzed by fluorescence detection after RP-HPLC separation. Experimental details are described in the Supplementary Information.

### 2.6. Pyrophosphate Hydrolysis by Human Nudt7

dpCoA, C2a, C3a, and C3b were pre-incubated with one equivalent TCEP, then incubated with Nudt7 at 37 °C and 300 rpm shaking for defined time intervals. After protein denaturation at 95 °C for 5 min and 10 kDa cutoff-filtration, thiol compounds were incubated with CPM dye (Abcam, Cambridge, UK, stock solution in dimethyl sulfoxide). Thiol-CPM conjugates were analyzed by fluorescence detection after RP-HPLC separation. Experimental details are described in the Supplementary Information.

### 2.7. HEK 293T Cell Experiments

A total of 10′000 HEK 293T cells were seeded in 100 µL medium on day 1. An amount of 500 µM HoPan and/or varying concentrations of PPanSH or C1 were added on day 2. Viable cells were quantified by the CellTiter 96 AQueous One Solution Cell Proliferation Assay (Promega) on day 5. Experimental details are described in the Supplementary Information.

## 3. Results

### 3.1. Enzymatic Synthesis of Thiophosphate Analogs

**Figure 1:**
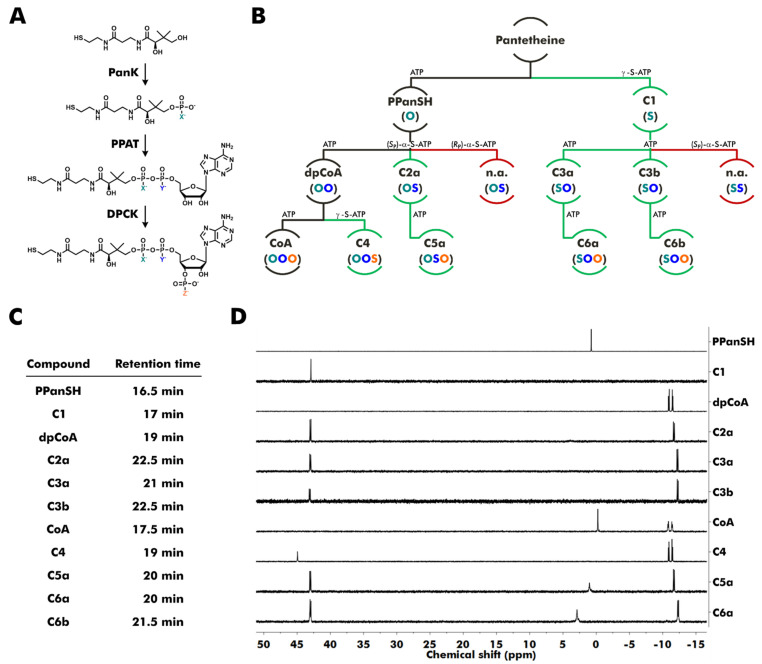
Synthesis of thiophosphate analogs of PPanSH, dpCoA, and CoA starting from pantetheine. (**A**,**B**) Reaction scheme for CoA and CoA thiophosphate analogs. The phosphorylation catalyzed by *E. coli* pantothenate kinase (PanK) yields PPanSH or a thiophosphate analog of PPanSH. The AMP transfer catalyzed by *E. coli* phosphopantetheine adenylyltransferase (PPAT) yields dpCoA or thiophosphate analogs of dpCoA. The phosphorylation catalyzed by *E. coli* dpCoA kinase (DPCK) yields CoA or thiophosphate analogs of CoA. (**B**) Colored letters in parentheses show the type of atom (oxygen or sulfur) in position X, Y, or Z of the respective compound shown in (**A**). Path descriptions display whether ATP or a thiophosphate analog of ATP was used to produce the respective compound. Black lines show the canonical path. Green lines show the paths to accessible novel products. Red lines show the paths to inaccessible products (n.a.: not accessible). (**C**) Approximate retention times of compounds during purification by reversed-phase high-performance liquid chromatography. (**D**) ^31^P nuclear magnetic resonance (NMR) spectra of purified molecules and commercially obtained CoA. Obtained amounts of C6b were too low to record a ^31^P NMR spectrum (ppm: parts per million).

The *Escherichia coli (E. coli)* biosynthetic enzymes pantothenate kinase (CoaA, PanK), phosphopantetheine adenylyltransferase (CoaD, PPAT), and dpCoA kinase (CoaE, DPCK) have been widely used to synthesize dpCoA, CoA, and several CoA derivatives with modified cysteamine or β-alanine moiety from pantetheine (Figure 1A) [19,20,21,22,23,24,25,26,27,28]. All three enzymes utilize adenosine triphosphate (ATP) to introduce either a phosphate group or an adenosine monophosphate (AMP) moiety. Here, ATP was exchanged by thiophosphate analogs of ATP, and acceptance by the enzymes was evaluated (Figure 1B). γ-S-ATP replaced ATP in the phosphorylation reactions catalyzed by *E. coli* PanK and *E. coli* DPCK. One of the two possible diastereomers of α-S-ATP, (*S*_P_)-α-S-ATP or (*R*_P_)-α-S-ATP, replaced ATP in the AMP transfer catalyzed by *E. coli* PPAT.

The reaction products were purified by preparative reversed-phase high-performance liquid chromatography (RP-HPLC) and analyzed by high-resolution mass spectrometry (HR-MS) and, if the isolated amounts were sufficient, by ^31^P nuclear magnetic resonance (NMR) spectroscopy (Figure 1C,D).

The phosphorylation of pantetheine by PanK in the presence of ATP or γ-S-ATP resulted in PPanSH or its thiophosphate analog, compound C1 (Figure 1B).

The PPAT-catalyzed reaction of PPanSH and ATP or (*S*_P_)-α-S-ATP produced dpCoA or compound C2a. Interestingly, PPAT accepted (*S*_P_)-α-S-ATP, whereas the usage of (*R*_P_)-α-S-ATP did not lead to any product formation. An in-line displacement mechanism was previously proposed for the reaction between PPanSH and ATP [29]. Consequently, there should be an inversion of the stereochemical configuration. Thus, the *R*_P_ configuration at the α-phosphorus is likely for C2a since (*S*_P_)-α-S-ATP was used in the synthesis. The PPAT-catalyzed reaction of C1 and ATP resulted in the simultaneous formation of two thiophosphate analogs of dpCoA: C3a and C3b. Both diastereomers were produced at a ratio of 1.4 to 1 and could be separated by RP-HPLC. Attempts to synthesize dpCoA analogs with two sulfur substitutions by a PPAT-catalyzed reaction from C1 and (*S*_P_)-α-S-ATP yielded no products. Thus, PPAT has a restricted substrate tolerance for thiosubstitutions: Substitution at the acceptor (PPanSH) produces a mixture of two diastereomers. Substitution at the α-position of the donor (ATP) is only accepted with *S*_P_ configuration, and the *R*_P_ isomer is inactive. A combination of thiosubstituted donor and acceptor is not tolerated at all (Figure 1B).

Similar to dpCoA, C2a, C3a, and C3b were also readily 3′-phosphorylated in the presence of ATP by the enzyme DPCK, resulting in the CoA thiophosphate analogs C5a, C6a, and C6b, which contain a modified pyrophosphate bridge. The reaction of dpCoA with γ-S-ATP yielded the desired CoA 3′-thiophosphate analog C4 (Figure 1B). Notably, all thiophosphate analogs exhibited increased retention times during RP-HPLC, indicating increased lipophilicity compared to their natural counterparts (Figure 1C).

### 3.2. Stability of Thiophosphate Analogs

**Figure 2:**
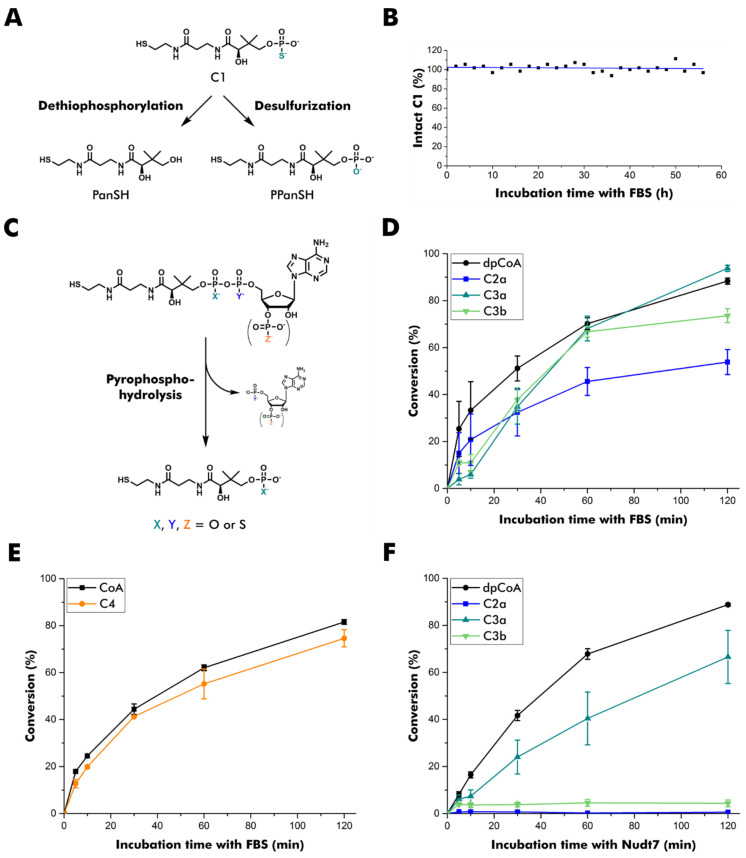
Stability of thiophosphate analogs of PPanSH, dpCoA, and CoA. (**A**) Overview of potential degradation pathways of C1. PanSH: pantetheine. (**B**) Stability of C1 in fetal bovine serum (FBS). The sample was monitored by ^31^P NMR spectroscopy. The signal of C1 was integrated and normalized to the phosphate peak arising from FBS. (**C**) Overview of pyrophosphate hydrolysis of dpCoA and CoA as well as thiophosphate analogs thereof. (**D**) Quantification of the conversion of dpCoA and C2a to PPanSH or C3a and C3b to C1 after incubation in FBS. (**E**) Quantification of the conversion of CoA and C4 to PPanSH after incubation in FBS. (**F**) Quantification of the conversion of dpCoA and C2a to PPanSH or C3a and C3b to C1 after incubation with human Nudt7. (**D**–**F**) Thiol compounds are quantified after conjugation to the fluorescence dye 7-diethylamino-3-(4-maleimidophenyl)-4-methyl-coumarin (CPM) and subsequent RP-HPLC separation. The peak heights of CPM conjugates were measured at 465 nm emission, and the product-to-substrate ratio was calculated. Error bars represent the standard deviation (n = 3).

In mammals, CoA is degraded to PPanSH by ectonucleotide pyrophosphatases in serum or by the Nudix-type hydrolases Nudt7, Nudt8, and Nudt19 intracellularly [11,12,13,30,31]. By contrast, PPanSH is known to be fairly stable [5,32]. Likewise, C1 proved to be stable to dethiophosphorylation and desulfurization in fetal bovine serum (Figure 2A,B, Figure S1). The effect of different thiophosphate modifications on CoA pyrophosphatases was studied subsequently (Figure 2C). Since C5a, C6a, and C6b were prepared in insufficient yield, their precursor molecules on the dpCoA level were used instead in these experiments. Potential substrates were incubated with fetal bovine serum or human Nudt7, and the conversion was analyzed after conjugation to a fluorescent dye followed by RP-HPLC separation (Figure S2) [33].

None of the thiophosphate modifications in the pyrophosphate bridge conferred full stability in fetal bovine serum, even though C2a degraded somewhat more slowly (Figure 2D). The degradation of C4 was comparable to that of CoA (Figure 2E), indicating that a 3′-thiophosphate group does not confer higher stability to pyrophosphate hydrolysis. Therefore, direct hydrolysis of CoA and C4 to PPanSH without prior 3′-de(thio)phosphorylation is likely to dominate the degradation process.

Human Nudt7 showed a more differentiated profile in vitro *(*Figure 2F). C2a was resistant to degradation, as was C3b. Interestingly, C3a, the stereoisomer of C3b, was readily accepted and transformed into C1. As C3a and C3b differ only in the configuration of the thiophosphate in β-position, this position appears important for the acceptance by this peroxisomal CoA pyrophosphatase. 

### 3.3. C1 Treatment Rescues CoA-Deficient Cells

**Figure 3:**
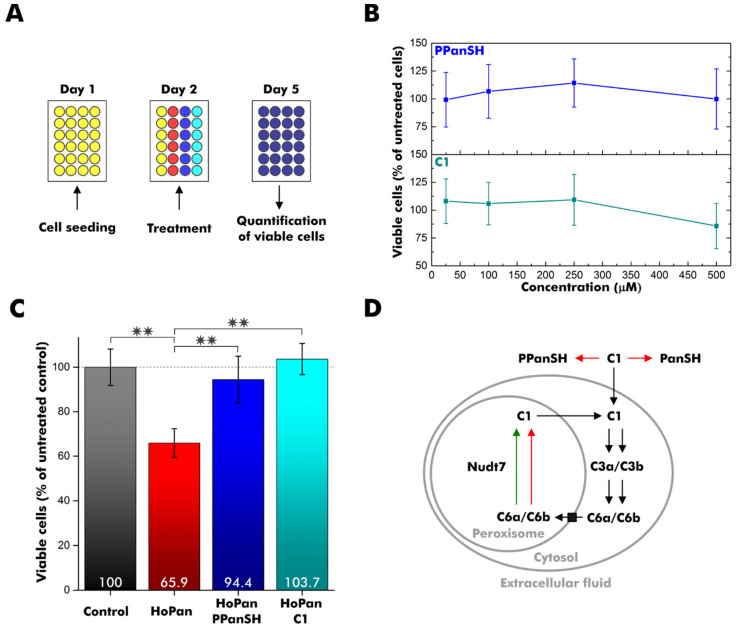
Supplementation of cell cultures with C1. (**A**) Experiment design overview. HEK 293T cells were seeded on day 1 and treated with calcium hopantenate (HoPan), PPanSH, C1, or a combination on day 2. Viable cells were quantified by a colorimetric cell proliferation assay on day 5. (**B**) Percentage of viable cells after growth in the presence of varying concentrations of PPanSH or C1 relative to untreated cells. (**C**) Percentage of viable cells after growth in the presence of 500 µM HoPan, 500 µM HoPan, and 25 µM PPanSH, or 500 µM HoPan and 25 µM C1 relative to untreated cells (control). The statistical analysis was carried out by two-sample *t*-test (n = 6), and double asterisks (**) represent a *p*-value < 0.01. (**B**,**C**) Error bars represent the standard deviation (n = 6). (**D**) Simplified scheme for the intracellular fate of C1. Red arrows indicate non-conversion. The green arrow indicates conversion. Black arrows are still hypothetical. PanSH: pantetheine.

In previous reports, supplementation with PPanSH was well-tolerated and could restore intracellular CoA levels of mammalian cells with impaired *de novo* CoA biosynthesis from pantothenate [5,10]. Since compound C3b had an increased stability against Nudt7 degradation, we investigated whether its biosynthetic precursor C1, the thiophosphate analog of PPanSH, still exhibits comparable properties in terms of biocompatibility and alleviation of CoA deficiency in a cell culture model. To study the cytotoxicity of C1, 10,000 human embryonic kidney (HEK 293T) cells were seeded on day 1 followed by a compound treatment on day 2 (Figure 3A and Figure S3A). The number of viable cells was quantified on day 5 using a cell proliferation assay. Although this assay is noted to interfere with excessive concentrations of thiol compounds, our workflow proved to be robust [34]. Background signals were only observed directly after the addition of PPanSH, but not for measurements after three days of incubation (Figure S3B).

Treatment of HEK 293T cells with increasing amounts of C1 revealed no acute toxicity at concentrations up to 250 µM (Figure 3B).

To probe the rescue potential, CoA deficiency was induced in HEK 293T cells using 500 µM calcium hopantenate (HoPan), which inhibits the conversion of pantothenate by the enzymes PANK and phosphopantothenoylcysteine synthetase [18,35]. The treatment reduced the viable cell count by approximately one-third (Figure 3C). To validate our disease model, HEK 293T cells were simultaneously incubated with 500 µM HoPan and 25 µM PPanSH. As observed in previous studies, PPanSH efficiently antagonized the negative effect of HoPan (Figure 3C) [5]. Intriguingly, comparable recovery was achieved by supplementation with 25 µM **C1** instead of PPanSH (Figure 3C). The viable cell count was almost identical to that of control cells without HoPan treatment. Consequently, C1 supported the intracellular CoA circuit in a hitherto unknown manner.

## 4. Discussion

In conclusion, we synthesized eight novel analogs of PPanSH, dpCoA, and CoA with one oxygen-to-sulfur modification in any of the phosphate groups present using the enzymes from the *E. coli* CoA biosynthesis pathway (Figure S4). In contrast to all dpCoA and CoA thiophosphate analogs tested, compound C1 (designated P_S_PanSH) was stable in serum and was able to restore normal cell proliferation after HoPan treatment. So far, we can only speculate on its mechanism of action. Membrane diffusion, conversion to the CoA thiophosphate analogs C6a and/or C6b by COASY, and subsequent execution of CoA-typical tasks may provide one possible hypothesis (Figure 3D). The observed acceptance of C3a by peroxisomal Nudt7 in vitro is likely to extend to its 3′-phosphorylated successor C6a and could introduce the latter into an intracellular recycling pathway, just as it is assumed for CoA (Figure 3D) [36]. On the other hand, C3b was not degraded by Nudt7 in vitro, suggesting peroxisomal accumulation of its 3′-phosphorylated successor C6b if formed in cellulo. Future studies should address the intracellular fate of P_S_PanSH and its successors to eventually infer its therapeutic applicability for genetic disorders affecting early CoA biosynthesis, PKAN in particular.

## Data Availability

Not applicable.

## Supplementary Materials

The following supporting information can be downloaded at: https://www.mdpi.com/article/10.3390/biom12081065/s1, Experimental Details; Figure S1: Stability of C1 in fetal bovine serum (FBS); Figure S2: Detection of thiol compounds by fluorescence; Figure S3: Cell proliferation assay optimization and validation; Figure S4: Overview of synthesized compounds.
