# Supplementary material for: Thiophosphate Analogs of Coenzyme A and Its Precursors—Synthesis, Stability, and Biomimetic Potential
Source: Biomolecules. 2022 Aug 1;12(8):1065. doi: 10.3390/biom12081065 (PMC9405834; doi:10.3390/biom12081065)
Supplement: Supplementary file 1 [file biomolecules-12-01065-s001.zip › biomolecules-1815110-supplementary-done.pdf]

biomolecules

Supplementary Information

Thiophosphate analogs of coenzyme A and its precursors – Synthesis, stability, and biomimetic potential

Christian Löcherer [1], Elif Tosun [1], Hannah Backes [1], and Andres Jäschke [1,*]

[1] Institute of Pharmacy and Molecular Biotechnology, Heidelberg University, Im Neuenheimer Feld 364, Heidelberg, 69120, Germany; c.loecherer@gmx.de; e.tosun@gmx.net; hannah.backes@t-online.de
* Correspondence: jaeschke@uni-hd.de; Tel.: +49-6221-544851

Citation: Löcherer, C.; Tosun, E.; Backes, H.; Jäschke, A. Thiophosphate Analogs of Coenzyme A and Its Precursors—Synthesis, Stability, and Biomimetic Potential. *Biomolecules* **2022**, *12*, 1065. https://doi.org/10.3390/biom12081065

Academic Editor: Vladimir N. Uversky

Received: 28 June 2022
Accepted: 29 July 2022
Published: 1 August 2022

Publisher's Note: MDPI stays neutral with regard to jurisdictional claims in published maps and institutional affiliations.

Copyright: © 2022 by the authors. Licensee MDPI, Basel, Switzerland. This article is an open access article distributed under the terms and conditions of the Creative Commons Attribution (CC BY) license (https://creativecommons.org/licenses/by/4.0/).

1. Table of Contents

Experimental Details

Supporting Figures

- **Figure S1:** Stability of C1 in fetal bovine serum (FBS).
- **Figure S2:** Detection of thiol compounds by fluorescence.
- **Figure S3:** Cell proliferation assay optimization and validation.
- **Figure S4:** Overview of synthesized compounds.

References

1. Experimental Details

General

All reagents were purchased from Sigma Aldrich (Schnelldorf, Germany) unless otherwise stated. Chemicals were generally of reagent grade or ACS grade. Solvents were generally of HPLC grade or molecular biology grade. Reagents were generally of molecular biology grade. Ultrapure water was obtained from a Milli-Q Advantage A10 Water Purification System (Merck Millipore, Darmstadt, Germany). All Kits and enzymatic reactions were performed according to manufacturer instructions unless otherwise stated. Nuclear magnetic resonance (NMR) spectra were recorded at a Mercury plus 300 MHz spectrometer (Varian, Palo Alto, CA, USA) or a Mercury plus 500 MHz spectrometer (Varian). Mass spectrometry (MS) spectra were recorded at a micrOTOF-Q II ESI mass spectrometer (Bruker Daltonics, Bremen, Germany).

Protein expression and purification

pET28a-Ec.coaA was a gift from Erick Strauss (Addgene plasmid # 50386). pET28a-Ec.coaD (pESC106) was a gift from Tadhg Begley & Erick Strauss (Addgene plasmid

50388). pET28a-Ec.coaE (pESC124) was a gift from Tadhg Begley & Erick Strauss (Addgene plasmid # 50390). NUDT7 (NUDT7A-c002) was a gift from Nicola Burgess-Brown (Addgene plasmid # 98235). Plasmids encoding for *E. coli* pantothenate kinase (PanK, coaA), *E. coli* phosphopantetheine adenylyltransferase (PPAT, coaD), *E. coli* dpCoA kinase (DPCK, coaE) and human Nudix-type hydrolase Nudt7 were isolated with the GeneJET Plasmid Miniprep Kit (ThermoFisher Scientific, Dreieich, Germany) and transformed into BL21(DE3) competent *E. coli* (ThermoFisher Scientific). 1 l of bacterial culture was grown to an OD_{600} of 0.6 – 0.8 at 37 °C and 165 rpm shaking. Expression of proteins with N-terminal 6x His-tags was induced by the addition of 1 ml of 1 M isopropyl β-D-1-thiogalactopyranoside (IPTG). After 3 h, the bacteria were pelleted and washed with 15 ml Dulbecco's phosphate-buffer saline. The pellet was resuspended in FPLC buffer A (300 mM NaCl, 1 mM imidazole, 50 mM Tris HCl pH 8, and 5% (V/V) glycerol) and cells were lysed by sonication. Cell debris was pelleted by ultracentrifugation. The filtrated supernatant was subjected to fast protein liquid chromatography (FPLC). Samples were loaded onto a HisTrap HP 1 ml column (GE Healthcare, Freiburg, Germany) in an NGC chromatography system (Bio-Rad, Feldkirchen, Germany). Elution was performed with a gradient from 3% to 100% FPLC buffer B (300 mM NaCl, 300 mM imidazole, 50 mM Tris HCl pH 8 and 5% (V/V) glycerol). Eluted fractions were analyzed by 12% SDS-PAGE and Coomassie staining. For PanK, PPAT, and DPCK, the pure protein fractions of the correct size were combined, and washed with storage buffer (300 mM NaCl, 50 mM Tris HCl pH 8, and 5% (V/V) glycerol) and concentrated by 10 kDa filtration. Concentrations were determined by UV absorbance at 280 nm on Nanodrop One (ThermoFisher Scientific) and the proteins were stored in 50% glycerol. For Nudt7, an additional size-exclusion purification on a Superdex 200 10/300 GL column (GE Healthcare) was conducted isocratically in the storage buffer. Protein fractions were again analyzed by 12% SDS-PAGE and Coomassie staining. Pure protein fractions of the correct size were combined and concentrated by 10 kDa filtration. Concentrations were determined by the BCA assay (ThermoFisher Scientific) and the proteins were stored in 50% glycerol.

Synthesis of 4′-phosphopantetheine (PPanSH)

150 µmol pantethine (2 eq.), 150 µmol tris(2-carboxyethyl)phosphine (TCEP, 2 eq.), and 151 µmol ATP disodium salt (1 eq.) were incubated with 10 nmol *E. coli* PanK in 5 ml of 20 mM KCl, 20 mM $MgCl_2$, 300 mM Tris HCl pH 7.5 at 37 °C and 300 rpm shaking for 15 h. Purification was performed by preparative RP-HPLC as described below. 84 µmol PPanSH were obtained as triethylammonium (TEA) salt (56% yield).

HR-MS (ESI-TOF, negative) m/z for $C_{11}H_{23}N_2O_7PS$ $[M-H]^-$, calculated: 357.0891, found: 357.0902.

^{31}**P NMR** (202 MHz, deuterium oxide (D_2O)) δ 0.74 .

Synthesis of compound C1 (S-PPanSH)

219 µmol pantethine (4.4 eq.), 261 µmol TCEP (5.2 eq.), and 100 µmol γ-S-ATP tetralithium salt (1 eq., Jena Bioscience, Jena, Germany) were incubated with 50 nmol *E. coli* PanK in 12 ml of 20 mM KCl, 20 mM $MgCl_2$, 300 mM Tris HCl pH 7.5 at 37 °C and 300 rpm shaking for 15 h. Purification was performed by preparative RP-HPLC as described below. 94 µmol of C1 were obtained as TEA salt (94% yield).

HR-MS (ESI-TOF, negative) m/z for $C_{11}H_{23}N_2O_6PS_2$ $[M-H]^-$, calculated: 373.0662, found: 373.0659.

^{1}**H NMR** (500 MHz, D_2O) δ 4.08 (s, 1H), 3.78 (dd, *J* = 10.1, 7.2 Hz, 1H), 3.53 – 3.44 (m, 3H), 3.35 (t, *J* = 6.6 Hz, 2H), 2.63 (t, *J* = 6.6 Hz, 2H), 2.50 (t, *J* = 6.6 Hz, 2H), 0.97 (s, 3H), 0.86 (s, 3H).

^{13}**C NMR** (126 MHz, D_2O) δ 174.69 , 173.95 , 74.70 , 70.85 , 42.24 , 38.20 (d, *J* = 8.2 Hz), 35.39 (d, *J* = 10.7 Hz), 23.05 , 21.13 , 18.47 .

^{31}**P NMR** (202 MHz, D_2O) δ 44.87 .

Synthesis of dpCoA

48 µmol pantethine (0.5 eq.), 75 µmol TCEP (0.75 eq.), and 400 µmol ATP disodium salt (4 eq.) were incubated with 3 nmol *E. coli* PanK and 4 nmol *E. coli* PPAT in 5 ml of 20 mM KCl, 20 mM $MgCl_2$, 300 mM Tris HCl pH 7.5 at 37 °C and 300 rpm shaking for 15 h. Purification was performed by preparative RP-HPLC as described below. 34.5 µmol dpCoA were obtained as TEA salt (36% yield).

HR-MS (ESI-TOF, negative) m/z for $C_{21}H_{35}N_7O_{13}P_2S$ $[M-H]^-$, calculated: 686.1416, found: 686.1422.

^{31}**P NMR** (202 MHz, D_2O) δ -10.93 (d, *J* = 21.4 Hz), -11.49 (d, *J* = 21.4 Hz).

Synthesis of C2a

66 µmol PPanSH (2.6 eq.) and 25 µmol (S_P)-α-S-ATP (1 eq., Biolog, Bremen, Germany) were incubated with 8 nmol *E. coli* PPAT in 5 ml of 20 mM KCl, 20 mM $MgCl_2$, 300 mM Tris HCl pH 7 at 37 °C and 300 rpm shaking for 15 h. Purification was performed by preparative RP-HPLC as described below. 9.4 µmol C2a were obtained as TEA salt (38% yield).

HR-MS (ESI-TOF, negative) m/z for $[M-H]^-$, calculated: 702.1188, found: 702.1261.

^{31}**P NMR** (202 MHz, Methanol-d_4) δ 44.33 (d, *J* = 32.5 Hz), -11.67 (d, *J* = 32.5 Hz).

The synthesis of C2b was attempted analogously except for the usage of (R_P)-α-S-ATP (Biolog) instead of (S_P)-α-S-ATP.

Synthesis of C3a and C3b

15.7 µmol C1 (1 eq.), 15.7 µmol TCEP (1 eq.), and 31.4 µmol ATP (2 eq.) were incubated with 2.5 nmol *E. coli* PPAT in 1 ml of 20 mM KCl, 20 mM $MgCl_2$, 300 mM Tris HCl pH 7.5 at 37 °C and 300 rpm shaking for 15 h. Purification was performed by preparative RP-HPLC as described below. Two distinct peaks at retention times of 21 min and 22.5 min were obtained. The fractions were referred to as C3a (21 min) and C3b (22.5 min).

1.1 µmol of C3a and 0.8 µmol of C3b were obtained as TEA salt (12.1% combined yield). C3a, **HR-MS** (ESI-TOF, negative) m/z for $C_{21}H_{35}N_7O_{12}P_2S_2$ $[M-H]^-$, calculated: 702.1188, found: 702.1186.

C3a, ^{31}**P NMR** (202 MHz, D_2O) δ 42.99 (d, *J* = 28.3 Hz), -12.21 (d, *J* = 28.3 Hz).

C3b, **HR-MS** (ESI-TOF, negative) m/z for $C_{21}H_{35}N_7O_{12}P_2S_2$ $[M-H]^-$, calculated: 702.1188, found: 702.1184.

C3b, **^{31}P NMR** (202 MHz, D_2O) δ 43.09 (d, *J* = 29.8 Hz), -12.22 (d, *J* = 29.5 Hz).

The synthesis of dpCoA thiophosphate analogs with two sulfur substitutions was attempted analogously except for the usage of (S_P)-α-S-ATP (Biolog) instead of ATP disodium salt.

Synthesis of C4

10 µmol dpCoA (1 eq.), 10 µmol TCEP (1 eq.), and 30 µmol γ-S-ATP (3 eq.) were incubated with 10 nmol *E. coli* DPCK in 1 ml of 20 mM KCl, 20 mM $MgCl_2$, 300 mM Tris HCl pH 8 at 37 °C and 300 rpm shaking for 15 h. Purification was performed by preparative RP-HPLC as described below. 2.76 µmol of C4 were obtained as TEA salt (27.6% yield).

HR-MS (ESI-TOF, negative) m/z for $C_{21}H_{36}N_7O_{15}P_3S_2$ $[M-H]^-$, calculated: 782.0851, found: 782.0895.

^{31}P NMR (202 MHz, D_2O) δ 44.93, -10.90 (d, *J* = 20.6 Hz), -11.46 (d, *J* = 20.8 Hz).

Synthesis of C5a

10 µmol C2a (1 eq.), 10 µmol TCEP (1 eq.), and 12 µmol ATP disodium salt (1.2 eq.) were incubated with 2 nmol *E. coli* DPCK in 1 ml of 20 mM KCl, 20 mM $MgCl_2$, 300 mM Tris HCl pH 8 at 37 °C and 300 rpm shaking for 15 h. Purification was performed by preparative RP-HPLC as described below. 3.14 µmol of C5a were obtained as TEA salt (31.4% yield).

HR-MS (ESI-TOF, negative) m/z for $C_{21}H_{36}N_7O_{15}P_3S_2$ $[M-H]^-$, calculated: 782.0851, found: 782.0821.

^{31}P NMR (202 MHz, D_2O) δ 43.01 (d, *J* = 28.5 Hz), 1.01, -11.69 (d, *J* = 28.6 Hz).

Synthesis of C6a

5 µmol C3a (1 eq.), 5 µmol TCEP (1 eq.), and 12 µmol ATP (2.4 eq.) were incubated with 2 nmol *E. coli* DPCK in 1 ml of 20 mM KCl, 20 mM $MgCl_2$, 300 mM Tris HCl pH 8 at 37 °C and 300 rpm shaking for 15 h. Purification was performed by preparative RP-HPLC as described below. 0.8 µmol of C6a were obtained as TEA salt (16% yield).

HR-MS (ESI-TOF, negative) m/z for $C_{21}H_{36}N_7O_{15}P_3S_2$ $[M-H]^-$, calculated: 782.0851, found: 782.0836.

^{31}P NMR (202 MHz, D_2O) δ 42.99 (d, *J* = 27.6 Hz), 2.89, -12.31 (d, *J* = 27.2 Hz).

Synthesis of C6b

0.5 µmol C3b (1 eq.), 0.5 µmol TCEP (1 eq.), and 0.6 µmol ATP (1.2 eq.) were incubated with 2 nmol *E. coli* DPCK in 1 ml of 20 mM KCl, 20 mM $MgCl_2$, 300 mM Tris HCl pH 8 at 37 °C and 300 rpm shaking for 15 h. Purification was performed by preparative RP-HPLC as described below. The obtained amount was too low to determine the yield and perform ^{31}P NMR characterization. Nevertheless, the desired product was identified by HR-MS.

HR-MS (ESI-TOF, negative) m/z for $[M-H]^-$, calculated: 782.0851, found: 782.0807.

Preparative reversed-phase high-performance liquid chromatography (RP-HPLC)

Reaction mixtures were filtrated through 10 kDa centrifugal filters. Purification was performed by preparative RP-HPLC using Agilent 1100 Series modules (Agilent Technologies, Waldbronn, Germany) and a Luna 5u C_{18} (250 mm x 15 mm, 5 µM, 100 Å, Phenomenex, Aschaffenburg, Germany) column. Separation was performed in a solvent system of 100 mM triethylammonium acetate (TEAA) pH 7 (solvent A) and 50 mM TEAA pH 7 in acetonitrile 1:4 (V/V) (solvent B) with a linear gradient from 2% B to 100% B in 98 min at a flow rate of 6 ml/min and UV detection at 230 nm and 260 nm. Peaks were collected and lyophilized. Products were dissolved in 5 ml ultrapure water and lyophilized once more to remove volatile buffer components. Compounds were obtained as triethylammonium (TEA) salts.

Synthesis of calcium hopantenate (HoPan)

The synthesis was performed as previously published [1]. 3.3 mmol 4-aminobutyric acid (1.1 eq.) were suspended in 10 ml methanol at room temperature and 3.3 mmol diethylamine (1.1 eq.) were added. 3 mmol (R)-pantolactone (1 eq.) were added and the mixture was stirred at 60 °C for 18 h. The solvent was removed *in vacuo*. The residual was dissolved in 20 ml deionized water and purified by an Amberlite IR-120 (H^+) ion-exchange column (2 cm x 10 cm). The eluate was extracted three times with 60 ml dichloromethane and the aqueous phase was evaporated to dryness. The residual was dissolved in 5 ml methanol and 0.84 mmol calcium hydroxide (0.6 eq.) were added. The mixture was stirred at 40 °C for 30 min. The solvent was evaporated *in vacuo* and the residual was dissolved in 3 ml ultrapure water and lyophilized. 252.4 mg product were obtained with a purity of 87%, as determined by ^{1}H NMR spectroscopy. Pantoate represented the major side product.

HR-MS (ESI-TOF, negative) m/z for $C_{10}H_{19}NO_5$ [M-H]$^-$, calculated: 232.1190, found: 232.1192.

^{1}H NMR (500 MHz, D_2O) δ 3.98 (s, 1H), 3.51 (d, *J* = 11.2 Hz, 1H), 3.39 (d, *J* = 11.3 Hz, 1H), 3.24 (t, *J* = 7.0 Hz, 2H), 2.24 (t, *J* = 7.6 Hz, 2H), 1.77 (p, *J* = 7.3 Hz, 2H), 0.93 (s, 3H), 0.90 (s, 3H).

Serum stability of C1

5 µmol C1 were incubated in a mixture of 75 µl 2x HEPES-buffered saline (274 mM NaCl, 5.4 mM KCl, 24 mM HEPES-Na, pH 7.4), and 75 µl D_2O and 600 µl dialyzed fetal bovine serum (FBS, ThermoFisher Scientific) at 37 °C. ^{31}P NMR spectra were recorded every 2 h for 56 h in total. After the 56 h-measurement, 5 µmol sodium thiophosphate hydrate (Carbosynth, Staad, Switzerland) were added and the sample was measured once more by ^{31}P NMR spectroscopy to ensure the separation of signals for C1 and thiophosphate.

50 µl of 100 mM PPanSH, 70 µl D_2O, and 580 µl dialyzed FBS (ThermoFisher Scientific) were mixed and measured by ^{31}P NMR spectroscopy to analyze the separation of chemical shifts for PPanSH and phosphate.

Pyrophosphate hydrolysis in fetal bovine serum (FBS)

The stability of different compounds (dpCoA, C2a, C3a, C3b, CoA, C4) in FBS was determined in triplicates as described in the following: 375 µl dialyzed FBS (ThermoFisher Scientific) was pre-incubated at 37 °C and 300 rpm shaking for 15 min. Then, 112.5 nmol compound of interest in 125 µl HEPES-buffered saline (HBS buffer, 137 mM NaCl, 2.7 mM KCl, 12 mM HEPES-Na, pH 7.4) were added and the reaction mixture was incubated at 37 °C and 300 rpm shaking. After defined intervals of 5 min, 10 min, 30 min, 60 min, and 120 min, aliquots of 100 µl were removed from the reaction mixture. Each aliquot was incubated at 95 °C for 5 min to stop the reaction, then cooled on ice. 45 µl of 1 mM TCEP and 5 µl 10x HBS buffer were added to each aliquot and the mixtures were incubated at room temperature for 15 min. The samples were further processed as described in below. For 0 min references, dialyzed FBS was heat-inactivated at 95 °C for 10 min and filtrated through a 10 kDa filter prior to the addition of the compound ofinterest.

Pyrophosphate hydrolysis by human Nudt7

The stability of different compounds (dpCoA, C2a, C3a, C3b) against pyrophosphate hydrolysis by human Nudt7 was determined in triplicates as described in the following: 90 nmol compound of interest and 90 nmol TCEP were incubated in 600 µl of 25 mM Tris HCl pH 7.5, 50 mM NaCl, 50 mM KCl, 10 mM $MgCl_2$ at room temperature for 15 min. 600 pmol Nudt7 were added and the reaction mixture was incubated at 37 °C and 300 rpm shaking. After defined intervals of 5 min, 10 min, 30 min, 60 min, and 120 min, aliquots of 120 µl were removed from the reaction mixture. Each aliquot was heated at 95 °C for 5 min to stop the reaction, then cooled on ice. The samples were further processed as described below. For 0 min references, the enzyme wasomitted.

CPM derivatization

After 10 kDa filtration of the samples of interest, 49.25 µl of the resulting filtrates were incubated with 0.75 µl of 10 mM 7-diethylamino-3-(4-maleimidophenyl)-4-methylcoumarin (CPM, Abcam, stock solution in dimethyl sulfoxide) at room temperature and light protection for 30 min. The CPM-derivatized thiols were analyzed by RP-HPLC using Agilent 1200 Series modules (Agilent Technologies) and a VDSpher PUR 100 C_{18}-SE (250 x 4.6 mm, 5 µm, VDS optilab, Berlin, Germany) column. Separation was performed in a solvent system of 100 mM TEAA pH 7 (solvent A) and 50 mM TEAA pH 7 in acetonitrile 1:4 (V/V) (solvent B) with a linear gradient from 2% B to 100% B in 40 min at a flow rate of 1 ml/min and fluorescence emission detection at 465 nm (excitation at 387 nm). Conversion rates were calculated based on the peak heights and normalized to 0 min references.

Background determination for the cell proliferation assay

On day 1, defined concentrations of PPanSH in 100 µl Dulbecco's Modified Eagle Medium (DMEM, ThermoFisher Scientific) supplemented with 10% FBS, and 1% penicillin-streptomycin were incubated in microplate wells at 37 °C and 5% CO_2 for 4 days. The absorption at 490 nm was measured on a Tecan Safire 2 microplate reader (Tecan, Männedorf, Switzerland) 2 h after the addition of 20 µl of CellTiter 96 AQueous One Solution Cell Proliferation Assay (Promega, Walldorf, Germany). Measurements were conducted on day 1 and day 4.

HEK 293T cell number optimization

On day 1, defined HEK 293T cell numbers in 100 µl DMEM (ThermoFisher Scientific) supplemented with 10% FBS, and 1% penicillin-streptomycin were seeded per microplate well and incubated at 37 °C and 5% CO_2. On days 2 – 5, viable cells were quantified at 490 nm absorption on a Tecan Safire 2 microplate reader (Tecan) 2 h after the addition of 20 µl of CellTiter 96 AQueous One Solution Cell Proliferation Assay (Promega).

Cell toxicity of C1

On day 1, 10'000 HEK 293T cells in 100 µl DMEM (ThermoFisher Scientific) supplemented with 10% FBS, and 1% penicillin-streptomycin were seeded per microplate well and incubated at 37 °C and 5% CO_2 for 5 days. On day 2, defined amounts of PPanSH or C1 or nothing were added. On day 5, viable cells were quantified at 490 nm absorption on a Tecan Safire 2 microplate reader (Tecan) 2 h after the addition of 20 µl of CellTiter 96 AQueous One Solution Cell Proliferation Assay (Promega). The absorption for treated cells was normalized to untreated cells.

HoPan treatment and recovery

On day 1, 10'000 HEK 293T cells in 100 µl pantothenate-free DMEM (Cell Culture Technologies, Gravesano, Switzerland) supplemented with 10% dialyzed FBS, and 1% penicillin-streptomycin were seeded per microplate well and incubated at 37 °C and 5% CO_2 for 5 days. On day 2, 50 nmol HoPan or 50 nmol HoPan and 2.5 nmol PPanSH or 50 nmol HoPan and 2.5 nmol C1 or nothing were added. On day 5, viable cells were quantified at 490 nm absorption on a Tecan Safire 2 microplate reader (Tecan) 2 h after the addition of 20 µl of CellTiter 96 AQueous One Solution Cell Proliferation Assay (Promega). The absorption for treated cells was normalized to untreated cells.

3. Supporting Figures

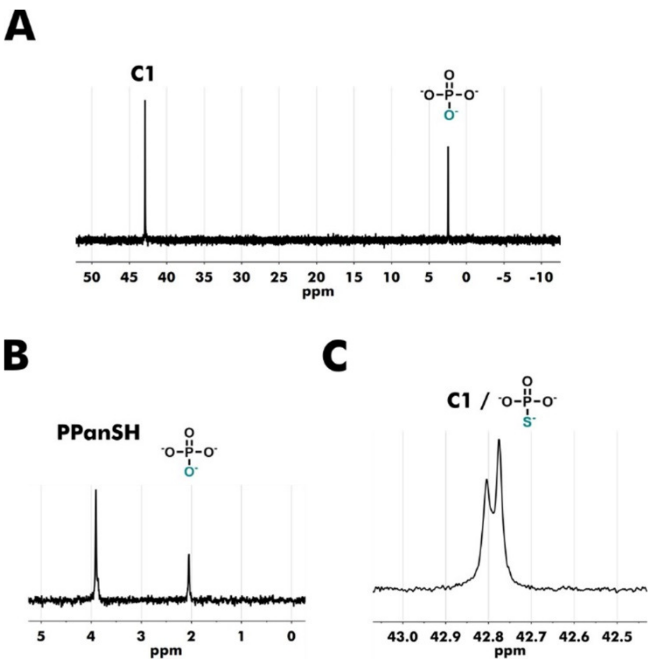

Figure S1. Stability of C1 in fetal bovine serum (FBS). **A** ^{31}P NMR spectrum of C1, after incubation in 80% FBS at 37 °C for 56 h. **B** ^{31}P nuclear magnetic resonance (NMR) spectrum of PPanSH, dissolved in 80% FBS, showing the separation of the PPanSH signal and the phosphate signal, which originated from FBS and served as a reference. **C** ^{31}P NMR spectrum of **A** after addition of thiophosphate, showing the separation of C1 and thiophosphate signals. **A** – **C** ppm: chemical shift in parts per million.

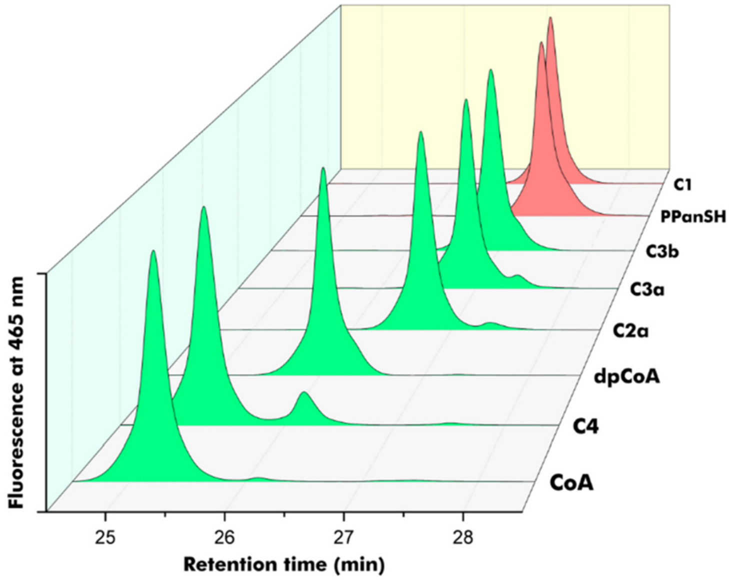

Figure S2. Detection of thiol compounds by fluorescence. RP-HPLC reference chromatograms of thiol compounds after conjugation to the fluorescence dye 7-diethylamino-3-(4-maleimidophenyl)-4-methyl-coumarin (CPM). Analyzed substrates of pyrophosphatases are displayed in green, products are displayed in red. Peak heights are normalized to the highest peak. Fluorescence was detected at 465 nm emission (excitation at 387 nm).

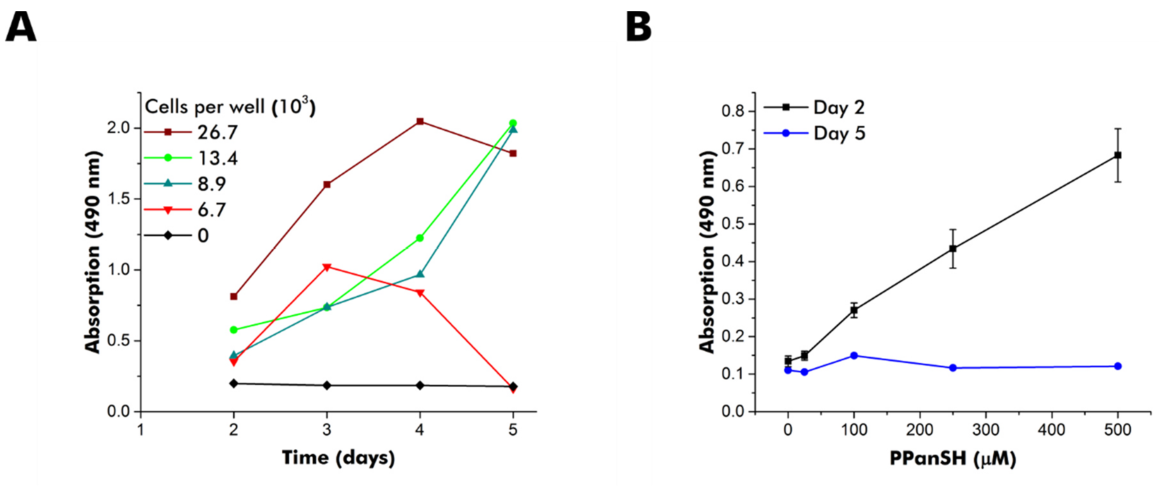

Figure S3. Cell proliferation assay optimization and validation. **A** Optimization of the seeded cell number for proper growth at day 5. A defined number of HEK 293T cells (Cells per well) was seeded on day 1 and the absorption at 490 nm caused by viable cells was recorded from day 2 to day 5 using the CellTiter AQueous One cell proliferation assay. **B** Assay robustness toward background due to the presence of thiol compounds. According to the standard workflow, thiols were added on day 2 and the cell proliferation was analyzed on day 5. Here, varying concentrations of PPanSH were directly quantified (day 2) or quantified after 3 days (day 5) by the CellTiter AQueous One cell proliferation assay. Viable cells were detected at 490 nm absorption. Error bars represent the standard deviation (n = 3).

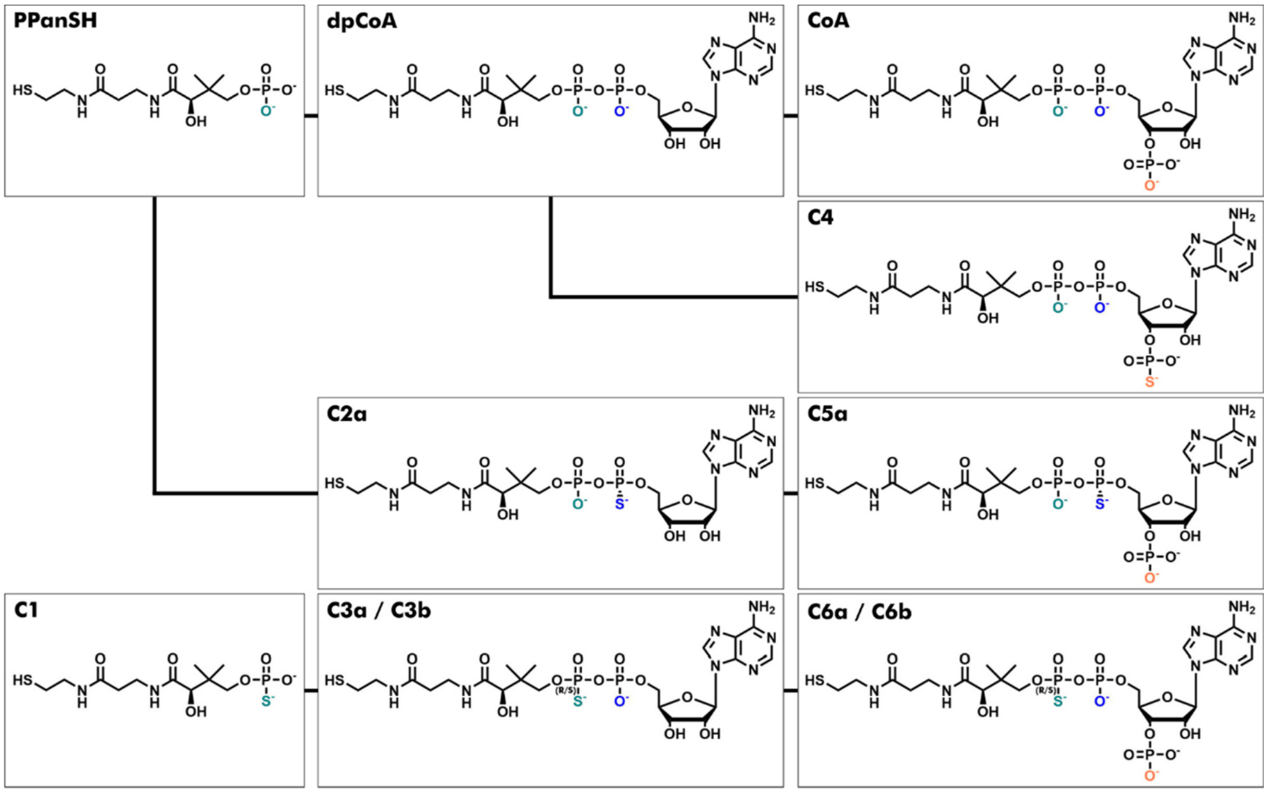

Figure S4. Overview of synthesized compounds. Connecting lines indicate precursor/successor molecules.

References

1. Zhang, Y.-M.; Chohnan, S.; Virga, K.G.; Stevens, R.D.; Ilkayeva, O.R.; Wenner, B.R.; Bain, J.R.; Newgard, C.B.; Lee, R.E.; Rock, C.O.; et al. Chemical Knockout of Pantothenate Kinase Reveals the Metabolic and Genetic Program Responsible for Hepatic Coenzyme A Homeostasis. *Chemistry & Biology* **2007**, *14*, 291-302, doi:https://doi.org/10.1016/j.chembiol.2007.01.013.
